# The protective effects of HIF-1α activation on sepsis induced intestinal mucosal barrier injury in rats model of sepsis

**DOI:** 10.1371/journal.pone.0268445

**Published:** 2022-05-16

**Authors:** Xiuzhen Lei, Wenbin Teng, Ying Fan, Yeke Zhu, Liuxu Yao, Yuhong Li, Shengmei Zhu

**Affiliations:** 1 Department of Anesthesiology, the First Affiliated Hospital, School of Medicine, Zhejiang University, Hangzhou, Zhejiang, China; 2 Department of Anesthesiology, Shaoxing People’s Hospital, Shaoxing University, Zhejiang, China; 3 Department of Anesthesiology, Shulan (Hangzhou) Hospital, Shulan International Medical College, Zhejiang Shuren University, Hangzhou, Zhejiang, China; Universidade Estadual de Ponta Grossa, BRAZIL

## Abstract

The integrity of the intestinal barrier is critical for protecting the host against the pathogen. The role of hypoxia-inducible factor-1α (HIF-1α) in the intestinal barrier disfunction related to sepsis remained unclear. The purpose of the present study is to investigate the role of HIF-1α on oxidative damage, the intestinal mucosal permeability, structural and morphological changes during sepsis. Twenty-four Sprague Dawley (SD) rats were randomly divided into four groups of 6 rats each: the sham group (sham), sepsis group (subjected to cecal ligation and perforation, CLP), sepsis + DMOG group (40 mg/kg of DMOG by intraperitoneal injection for 7 consecutive days before CLP), and sepsis + BAY 87–2243 group (9 mg/kg of BAY 87–2243 orally administered for 3 consecutive days before CLP). Sepsis increased plasma levels of inflammatory mediators, oxidative stress markers and HIF-1α expression; caused pathological damage; increased permeability (*P* < 0.05); and decreased TJ protein expression in the intestinal mucosa of rats with sepsis (*P* < 0.05). The addition of DMOG up-regulated HIF-1α, then decreased the plasma levels of inflammatory mediators, oxidative stress markers, alleviated pathological damage to the intestinal mucosa and decreased intestinal permeability (*P* < 0.05); while BAY 87–2243 treatment had the opposite effects. Our findings showed that HIF-1α protects the intestinal barrier function of septic rats by inhibiting intestinal inflammation and oxidative damage, our results provide a novel insight for developing sepsis treatment.

## Introduction

Sepsis, a life-threatening type of organ dysfunction caused by a maladjusted response to infection, is a recognized global health problem and serious threat to human life. Global statistical data presented in 2017 [1] reported 48.9 million sepsis cases and 1.1 million death, with a mortality rate of 19.7%; although guidelines for the treatment of sepsis provide some evidence-based medical advice for the clinical management of sepsis patients, many gaps in knowledge of the pathogenesis of sepsis remain [2, 3].

The causes and mechanisms of the occurrence, development and deterioration of sepsis are relatively complicated. Currently, it is widely believed that inflammation and oxidative stress injury, and immune dysfunction of the body are involved in the occurrence and development of sepsis and may be the main factors for the initiation of sepsis [4]. At present, the relationship between cytokines, inflammatory mediators, free radical injury, oxidative stress related to sepsis has attracted more and more attention. The intestinal mucosal barrier is an important organ for maintaining the stability of the internal environment. Damage to intestinal mucosal barrier function can cause intestinal bacterial translocation and enterogenous infection, and subsequent systemic inflammatory response syndrome, MOD, and even multiple organ failure (MOF) [5]. Considering this, In view of this, improving intestinal mucosal function may be one of the key problems to improve the prognosis of patients with sepsis.

In severe diseases and trauma, especially sepsis, the intestinal mucosal barrier is damaged to varying degrees [6]. The mechanism of intestinal mucosal barrier injury is very complicated and involves ischemia and hypoxia, physical and chemical immune factors, changes in the intestinal microflora, oxygen free radicals and other inflammatory mediators. Previous studies have shown that inflammatory factors and oxidative stress products are significantly increased when the intestinal mucosal barrier is damaged, and antioxidant treatment can reduce the damage to a certain extent [7]. However, further study regarding how oxygen free radicals damage the intestinal mucosal barrier remains necessary.

Hypoxia-inducible factor-1α (HIF-1α) is a key transcription factor through which the human body adapts to hypoxic environments and plays a key role in the acute hypoxia response [8], which is related to the pathophysiology of many major ischemic and hypoxic diseases and is known to be involved in sepsis and has already been proposed as both a marker and a therapeutic target of sepsis. In recent years, the impact of intestinal dysfunction on the outcome of sepsis has been a research focus. In the early stage of sepsis, the hypoxia-induced disturbance of intestinal epithelial cells metabolism is an important cause of intestinal dysfunction. Whether the body can increase metabolism, reduce the inflammation and oxidative stress response to address damage to the intestinal mucosal barrier in sepsis by upregulating HIF-1α expression is unclear. In the present study, DMOG, an inhibitor of prolyl hydroxylase, was used as a stimulator to upregulate HIF-1α expression, while BAY 87–2243 as a inhibitor to downregulate HIF-1α expression. The purpose of this study was to investigate the effect of HIF-1α on intestinal mucosal barrier injury in sepsis. We hypnoses that HIF-1α will present its protection effect on sepsis-induced intestinal mucosa injury through modulation of inflammatory and oxidative process. We wish the result to provide an experimental basis for the treatment of injury to intestinal mucosal barrier function in sepsis.

## Materials and methods

### Animals

Twenty-four SPF male Sprague Dawley (SD) rats weighing 200–250 g were provided by the Zhejiang Academy of Medical Sciences (license No. SCXK (Zhe) 2-14-0001). The experiment was completed in full within 20 days. The research scheme was discussed and approved by the Academic Committee and Animal Ethics Committee of Shaoxing People’s Hospital (ethical approval No. 2020–009). The experiment was completed in the animal experimental center of Shaoxing People’s Hospital (SYXK [Zhe] 2017–0007) in strict accordance with the standard operating procedures for the use of experimental animals set by Zhejiang University. The investigators who conducted the experiments had completed an Institutional Animal Care and Use Committee training course on animal care and handling and then obtained animal experiment license. The operations were carried out under anesthesia in an effort to reduce the pain of animals.

### Reagents and kits

Sevoflurane (Jiangsu Hengrui Pharmaceutical Co., Ltd., China), sufentanil citrate injection (Yichang Renfu Pharmaceutical Co., Ltd., China), ropivacaine hydrochloride (AstraZeneca Pharmaceutical Co., Ltd.), diamine oxidase (DAO), intestinal type fatty acid binding protein 2 (FABP2) kit (Cloud clone, USA), D-lactic acid kit (Abebio, Wuhan, China), fluorescein isothiocyanate-dextran (FD4) and lipopolysaccharide (LPS), rat IL-6, IL-10 and TNF-α ELISA kit (Shanghai Beyotime Company, China); malondialdehyde (MDA) kit (S0131, Shanghai Beyotime Company, China); superoxide dismutase (SOD) kit (Beijing Solarbio Company, China), catalase (CAT) kit (Bc0200, Beijing Solarbio Company, China). The first antibodies of HIF-1α, ZO-1, occludin, claudin-1, GAPDH (Abcam Company, USA), dimethylglyoxyl glycine (DMOG) and BAY 87–2243 were provided by MedChemExpress USA. Horseradish peroxidase (HRP)—labeled Goat anti rabbit IgG (second antibody, Jackson Company, USA). SpectraMax Plus, Shanghai Megi molecular Instrument Co., Ltd.; The SPARK Multimode Microplate Reader, Tecan, Switzerland; Gel imaging system (ChemiDoc XRS; American Company); Quantity one 4.6 image processing and analysis system (Chemidoc XRS, Biorad, USA); Animal anesthesia machine (R5110IP, Shenzhen Ruiwode Life Technology Co., Ltd. China).

### Establishment of sepsis model rats

SD rats were allowed to adapt to the environment 3 days with free access to water and fasted for 6 hours before the experiment. After blood pressure (BP) and heart rate (HR) were monitored by noninvasive sphygmomanometer (BP-17T, Beijing Ruanlong Biotechnology Co., Ltd. China) a cecal ligation and perforation (CLP) model was established as follows: All animals were anesthetized via 3%-5% sevoflurane inhalation with 50% fraction of inspired oxygen (FiO_2_) and the peritoneal injection of 0.1 μg/kg sufentanil during surgical procedures, and their body temperatures were maintained at 36–38°C with a heating pad. After the skin had been prepared and disinfected, a 1.5 cm incision was made through the midline. Subsequently, the cecum was ligated 0.5 cm below the cecum and proximal ileocecal valve with a No.1 suture and gently squeezed to extrude a small amount of feces from the perforation site. After the CLP procedure, warm Ringer’s solution (50 ml/kg) was injected subcutaneously for fluid supplementation, and 0.5% ropivacaine (1 ml/kg) was injected for postoperative analgesia. After the operation, the animal were placed back into their cages and allowed to eat and drink freely. Animal health and behavior were monitored carefully every 4 hrs until 24 hrs post-CLP, during which, the animals meeting one of the criteria (unconsciousness, lack of reactivity, labored breathing, and cyanosis) were euthanized by cervical dislocation.

### Grouping and modeling

Twenty-four SD rats were randomly divided into the sham group (sham), sepsis group (sepsis), sepsis + DMOG (using as HIF-1α activator) and sepsis + BAY 87–2243 (using as HIF-1α inhibitor) group, each of which contained 6 rats. CLP was used to establish a sepsis model. Rats in the sepsis + DMOG group were administered DMOG (40 mg/kg) by intraperitoneal injection for seven days before CLP, and those in the sepsis + BAY 87–2243 group were orally administered BAY 87–2243 (9 mg/kg) for 3 days before CLP. Rats in the sham and sepsis groups were given the same volume of normal saline containing 10% ethanol and 40% Solutol HS-15 by oral gavage. DMOG was dissolved in normal saline and stored at a concentration of 40 mg/ml. BAY 87–2243 was dissolved in normal saline containing 10% ethanol and 40% Solutol HS-15 and stored at 9 mg/ml. All reagents were stored at -20°C and dissolved before use. The randomization was as follows: all of rats were numbered in sequence and put into the cages in order. Twenty-four non repeating random integers were generated by using Excel. The rats corresponding to random numbers 1 to 6 were enrolled into sham group; the rats corresponding to random numbers 7 to 12 into sepsis group; the rats corresponding to random numbers 13 to 18 into sepsis + BMOG group; the rats corresponding to random numbers 19 to 24 into sepsis + BAY 87–2243 group.

### Specimen collection and disposal

At 24 hours post-CLP, under sevoflurane and sufentanil anesthesia, all animals underwent open surgery through the prior incision. First, the intestine of 4 cm was separated, the two ends of the intestinal tube were ligated with surgical sutures, and 50 μg of FD4 with concentration of 100 mg/ml was injected into the intestinal tube. 0.5 ml of blood was taken from the portal vein 1 h later for the determination of FD4 plasma concentration. Then, 2 ml of blood was taken from the abdominal aorta and placed in an EDTA anticoagulant tube. One hour later, the blood samples were centrifuged at 3000 rpm and 4°C for 10 min, after which the plasma was separated and stored at -80°C to assess intestinal mucosal permeability markers (DAO, FABP2, and D-lactic acid), inflammatory markers (IL-6, IL-10, and TNF-α), and oxidative stress indicators (SOD, CAT, and MDA levels). A few 1-cm segments of the ileum were taken 2 cm away from the cecum, and some of these segments were used to prepare paraffin blocks for morphological study, while the others were stored at -80°C and used to detect the expression TJ proteins and HIF-1α. Then, the rats were killed by cervical dislocation. The observer who did not know the protocol conducted outcome assessment, data record and data analysis.

### HE staining was used to detect morphological changes in the intestinal mucosa

After the paraffin-embedded blocks had been sectioned, stained with hematoxylin and erosin (HE) and sealed, the morphological changes in the small intestinal mucosa were observed under a light microscope, and the degree of small intestinal tissue damage was determined by calculating the Chiu score.

### Determination of the oxidative stress level

With an MDA detection kit, plasma MDA was detected based on the reaction of MDA with thiobarbituric acid (TBA), which produces a red product. The OD value of the MDA-TBA adduct at an excitation wavelength of 535 nm was determined, and a concentration standard curve was then established and used to calculate the plasma MDA concentration.

A SOD activity test kit was used according to the instructions to determine the level of plasma SOD by visible spectrophotometry. SOD can clear superoxide anion (O^2-^) and inhibit the formation of reactive oxygen species. O^2-^ is produced from xanthine by xanthine oxidase and can reduce nitroblue tetrazolium to form a blue product that absorbs at 560 nm.

A CAT activity test kit was used according to the instructions to determine the plasma CAT level by UV spectrophotometry, CAT can decompose H_2_O_2_, which has a characteristic absorption peak at 240 nm, in a time-dependent manner. CAT activity can then be calculated according rate at which the absorbance (*A*) changed.

### Detection of plasma DAO, FABP2, D-lactic acid and inflammatory mediator levels by enzyme-linked immunosorbent assay (ELISA)

The plasma expression levels of DAO, FABP2, D-lactic acid and inflammatory mediators (IL-1β, IL-6 and TNF-α) were detected with ELISA kits according to the instructions. The *A* at 450 nm was obtained with a microplate reader. A standard curve was drawn according to the *A*. The DAO, FABP2, D-lactic acid and inflammatory mediators contents in each sample were calculated, and the average values were taken.

### Determination of the FD-4 concentration

After thawing, the plasma was diluted 1:8 and transferred to 96 well plate. Each sample was placed into a double well, with each well containing 100 μl. The FD4 concentration was measured with a fluorescence multi-mode microplate reader. The excitation and emission wavelengths were set at 490 nm and 520 nm respectively. All experimental procedures were performed strictly according to the instructions.

### Western blot detection of TJ proteins and HIF-1α expression in the intestinal mucosa

After treatment, the tissues were washed twice with phosphate-buffered saline (PBS) supplemented with PMSF and RIPA and lysed on ice for 30 minutes. Then, the sample was centrifuged for 15 minutes, and the supernatant was extracted. After quantification by BCA assay, 40 g of total protein was mixed with an equal volume of 2 × sample buffer and boiled at 100°C for 3 min for denaturation. The sample was electrophoresed on a 6%-12% SDS-polyacrylamide gel. The proteins were routinely transferred to a PVDF membrane that was sealed with TBST containing 5% bovine serum albumin at room temperature for 2 hours. Then, the PVDF membrane was washed with TBST 3 times for 5 minutes each. Anti-HIF-1α (1:1000), anti-ZO-1 (1:1000), anti-Occludin (1:1000), anti-Claudin-1 (1:1000), and anti-GAPDH (1:1000) antibodies were added and incubated overnight at 4°C. After being fully washed with TBST, the PVDF membrane was intubated with peroxidase–conjugated affinipure goat anti-rabbit antibody (1:5000) at room temperature for 2 hours, washed 3 times and developed with an ECL luminescence kit. The membrane was photographed with a gel imaging system, and the gray values were analyzed by ImageJ software.

### Sample size determination

Sample size was estimated based on assuming a standard deviation (SD) of 10%-15% of the mean (based on previous studies). Power was given at 0.85 to detect a 20% difference in the mean between groups at *P* < 0.05 (two-tailed) with use of G*Power (Version 3.0.10, University of Kiel, Germany). Therefore, A total of 20 rats were calculated at the minimum sample size. To prevent the effects of dropout, one rat in each group was added, for a total of 24 rats.

### Statistical analysis

The data were analyzed with GraphPad prism (version 7.0). All experimental data are expressed as the mean (SD). One-way ANOVA was used to analyze the differences among groups. For data with a homogeneous distribution, LSD was used for pairwise comparison. For data with an inhomogeneous distribution, the Dunnett T3 test was used. The test level (α) was set to 0.05.

## Results

### General condition of the animals

All animals were tolerated the procedures well, their BP and HR maintained stable during the CLP procedure. Sepsis appeared 6 hours after CLP, as observed through clutching, decreased activity, mucus secretion, the presence of erect hair, etc. Behavioral changes in the rats were observed and recorded every 4 hours after CLP. At 24 hours after CLP, one rat in the sepsis + BAY 87–2243 group died, but the other animals survived.

### Effects of an HIF-1α activation/inhibition on intestinal mucosal morphology in septic rats

**Fig 1:**
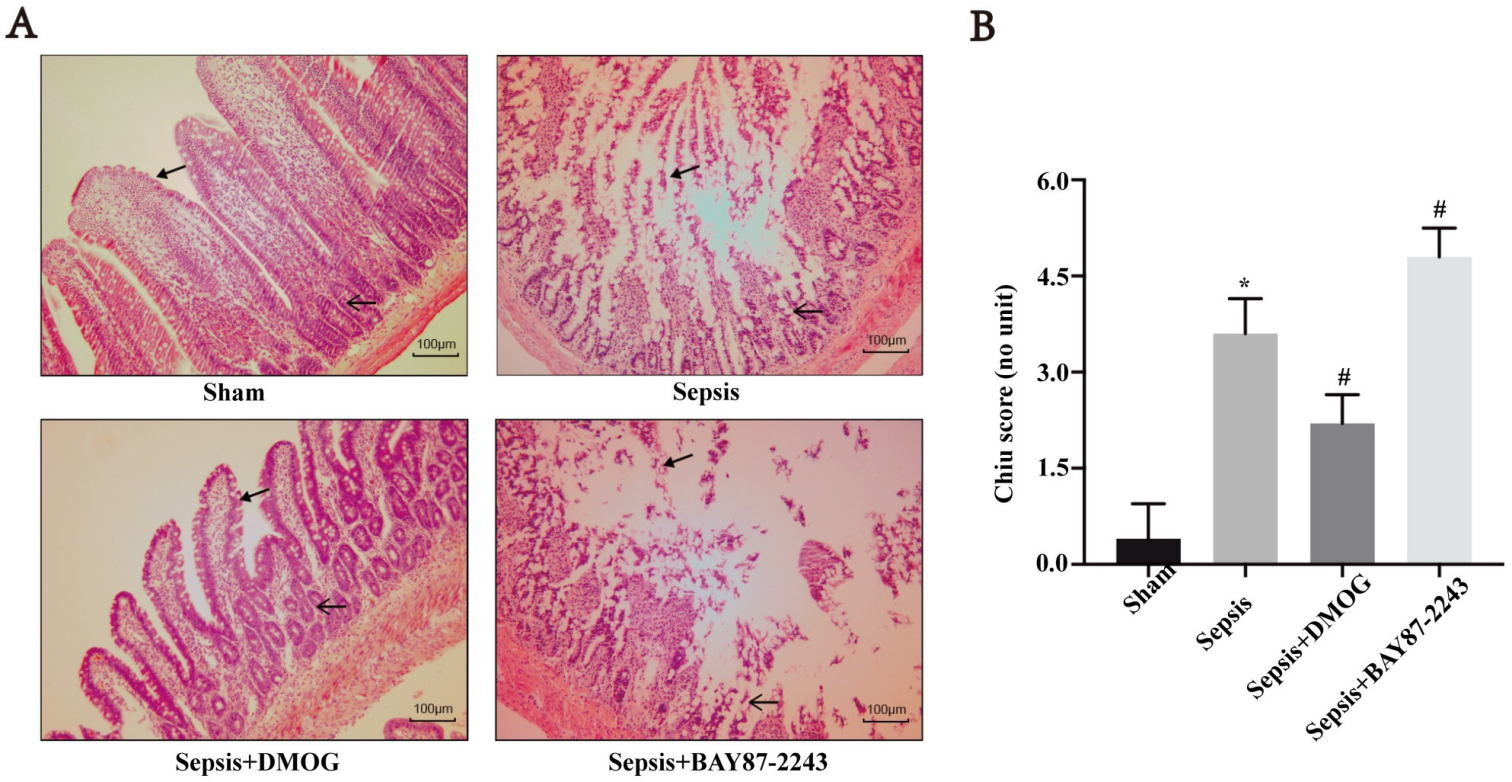
Effects of an HIF-1α activation/inhibition on the intestinal mucosa in septic rats, as shown by HE staining (×200) and the Chiu score of (*n* = 6 except sepsis + BAY 87–2243 with *n* = 5). Data are expressed as the mean (SD). A. Representative images showing the intestinal morphology (HE staining, magnification × 200). B. Intestinal mucosa injury was quantitatively assessed by calculating the Chiu score; *P < 0.05 compared with the sham group; ^#^*P* < 0.05 compared with the sepsis group.

In the sham group, a clear intestinal villi boundary with no hyperplasia of the lamina propria was observed (Fig 1A), while in the sepsis group, obvious proliferation of the lamina propria with a large number of intestinal villi epithelium protruding upward, shedding, and a significantly increased Chiu score (*P* < 0.05, Fig 1A and 1B). In the sepsis + DMOG group, the intestinal villi exhibited a clear boundary, no obvious intestinal epithelial shedding or necrosis was observed, obvious lamina propria hyperplasia was observed, and the Chiu score was significantly lower then that in the sepsis group (*P* < 0.05) (Fig 1A and 1B). In the sepsis + BAY 87–2243 group, the intestinal villi were disordered, the intestinal epithelium was obviously exfoliated and necrotic, the lamina propria was clear and ulcerated, and the Chiu score was significantly increased compared to that in the sepsis group (*P* < 0.05) (Fig 1A and 1B).

### Effects of an HIF-1α activation/inhibition on inflammation and oxidative stress in septic rats

**Fig 2:**
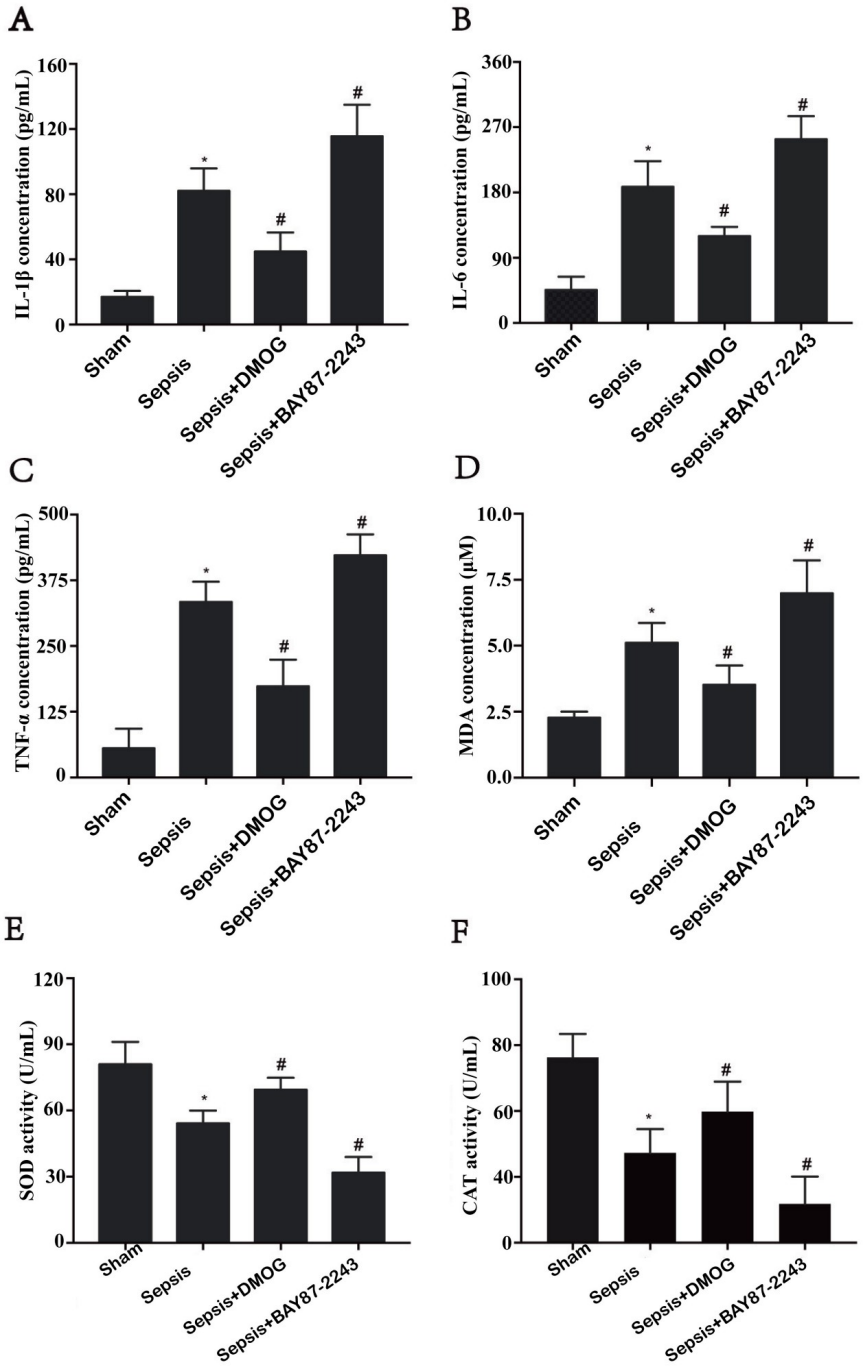
Effects of an HIF-1α activation/inhibition on inflammation and oxidative stress in septic rats (*n* = 6 except sepsis + BAY 87–2243 with *n* = 5). Data are expressed as the mean (SD). Changes in IL-6 (A), IL-1β (B), and TNF-α (C) plasma concentrations; the MDA plasma content (D); and SOD (E) and CAT (F) activities in each group are depicted. *P< 0.05 compared with the sham group; ^#^*P* < 0.05 compared with the sepsis group.

Compared with those in the sham group, the levels of IL-6, IL-1β and TNF-α in the sepsis group were increased (Fig 2A–2C, *P* < 0.05), the MDA content was increased, and the SOD and CAT activities were decreased (Fig 2D–2F, *P* < 0.05). Compared with those in the sepsis group, the levels of IL-6, IL-1β and TNF-α were decreased (Fig 2A–2C, *P* < 0.05), the MDA content was decreased, with the SOD and CAT activities increasing in the sepsis + DMOG group (Fig 2D–2F, *P* < 0.05). Compared with those in the sepsis group, the levels of IL-6, IL-1β and TNF-α were increased (Fig 2A–2C, *P* < 0.05), the MDA content was increased, while the SOD and CAT activities were decreased in the sepsis + BAY 87–2243 group (Fig 2D–2F, *P* < 0.05).

### Effects of an HIF-1α activation/inhibition on the plasma DAO, FABP2, D-lactic acid concentrations and FD4 permeability in septic rats

**Fig 3:**
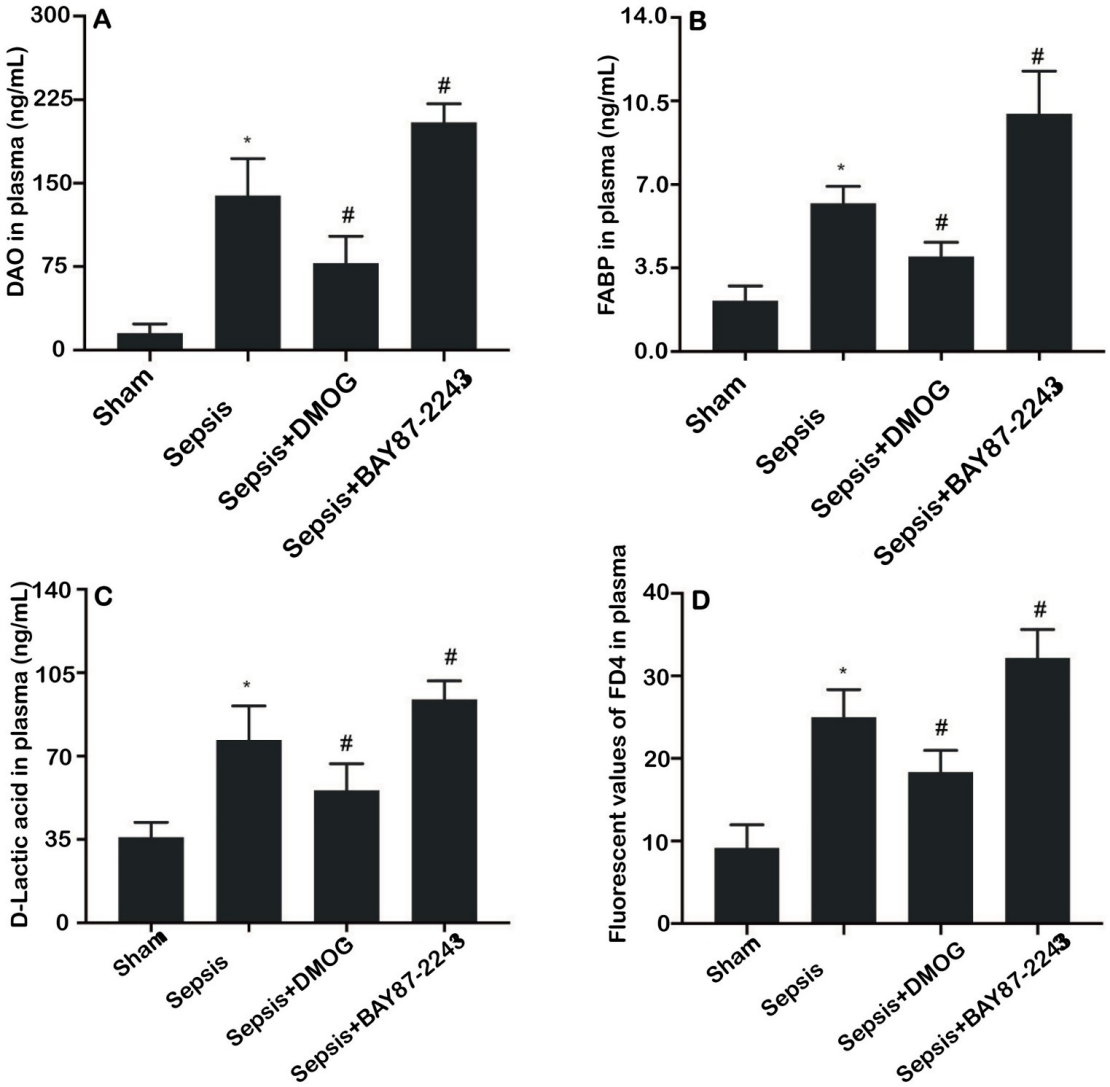
Effects of an HIF-1α activation/inhibition on the levels of DAO (A), FABP2 (B), and D-lactac acid (C) and the fluorescent signal corresponding to FD4 (D) in the plasma of rats (*n* = 6 except sepsis + BAY 87–2243 with *n* = 5). Data are expressed as the mean (SD); *P < 0.05 compared with the sham group; ^#^*P* < 0.05 compared with the sepsis group.

Compared with those in the sham group, the levels of DAO, FABP2, D-lactic acid and FD4 in the sepsis group were significantly increased. Additionally, compared with those in the sepsis group, the levels of DAO, FABP2, D-lactic acid and FD4 in the sepsis + DMOG group were significantly decreased (Fig 3A–3D) (*P* < 0.05), however, the levels of DAO, FABP2, D-lactic acid and FD4 in the sepsis + BAY 87–2243 group were increased (Fig 3A–3D; *P* < 0.05).

### Effects of an HIF-1α activation/inhibition on TJ proteins in the intestinal mucosa of septic rats

**Fig 4:**
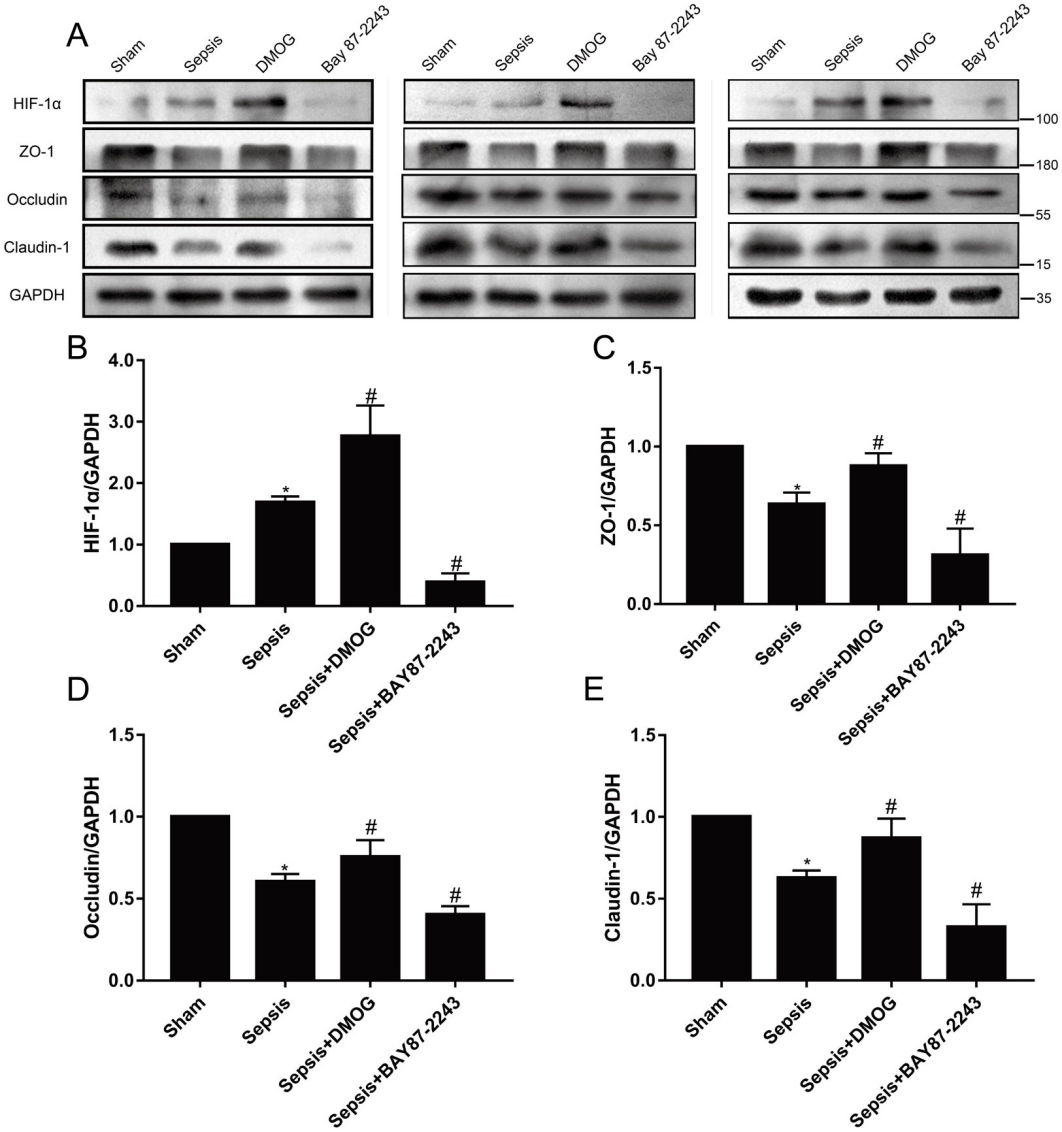
Effects of an HIF-1α activation/inhibition on the protein expression of HIF-1α (B) and TJ proteins (C-E) in the intestinal mucosa of rats (*n* = 6 except sepsis + BAY 87–2243 with *n* = 5). Data are expressed as the mean (SD). A. Bands showing protein expression (three animals). B-E. Protein expression of HIF-1α, ZO-1, Occludin, and Claudin-1. *P < 0.05 compared with the sham group; ^#^*P* < 0.05 compared with the sepsis group.

Compared with that in the sham group, the expression level of HIF-1α was significantly increased in the sepsis group (Fig 3A and 3B; *P* < 0.05), and the expression levels of ZO-1, occludin and claudin-1 were significantly decreased (Fig 4A and 4C–4E; *P* < 0.05). Compared with the sepsis group, the sepsis + DMOG group exhibited significantly higher expression levels of HIF-1α and ZO-1, occludin and Claudin-1 in the intestinal mucosa (Fig 3A and 3B; *P* < 0.05) (Fig 3A and 3C–3E; *P* < 0.05). Furthermore, the expression level of HIF-1α was lower in the sepsis + BAY 87–2243 group (Fig 4A and 4B; *P* < 0.05), and the protein expression levels of ZO-1, occludin and claudin-1 protein were significantly decreased (Fig 3A and 3C–3E and Table 1; *P* < 0.05).

**Table 1:** Effects of an HIF-1α activation/inhibition on the expression of HIF-1α and TJ proteins in the intestinal mucosa in rats.

Protein/reference	Sham (*n* = 6)	Sepsis (*n* = 6)	Sepsis + DMOG (*n* = 6)	Sepsis + BAY 87–2243 (*n* = 5)
HIF-1α/GAPDH	1.00 (0.00)	1.69 (0.10)*	2.77 (0.50)^#^	0.39 (0.14)^#^
ZO-1/GAPDH	1.00 (0.00)	0.63 (0.07)*	0.88 (0.08)^#^	0.31 (0.17)^#^
Occludin/GAPDH	1.00 (0.00)	0.62 (0.06)*	0.77 (0.14)^#^	0.38 (0.02)^#^
Claudin/GAPDH	1.00 (0.00)	0.63 (0.05)*	0.87 (0.12)^#^	0.33 (0.14)^#^

Data are expressed as the mean (SD);

*P < 0.05 compared with the sham group;

^#^*P* < 0.05 compared with the sepsis group.

## Discussion

Sepsis is a critical medical condition with rapid progression and high mortality. Intestinal mucosal dysfunction due to sepsis has been as an initiator of MOD [5, 6]. In the present study, CLP model of sepsis in the rat, we observed intestinal mucosal morphology and function, manifested by destructed intestinal mucosal structure and increased intestinal permeability. These alterations paralleled alterations in oxidative stress that we found significantly augmented lipid peroxidation along decreased activities of CAT and SOD and in inflammatory response that we observed the increased plasma concentration of inflammatory mediators.

Previous findings indicate a complex and dynamic relationship between intestinal mucosal permeability and immune homeostasis [9]. In sepsis, impaired intestinal mucosal function and increased intestinal permeability lead to the translocation of intestinal bacteria and endotoxins to the blood and abdominal cavity, aggravating the condition. D-lactate acid, a metabolite of intestinal bacteria, and DAO, an enzyme high in the intestinal mucosa of mammalian species, are sensitive markers for intestinal mucosal lesions and barrier function [10]. FD4 and FABP2 have also been proven to be the markers of changes in intestinal permeability and the intestinal barrier [11]. In the present study, the levels of plasma D-lactate acid, DAO, FABP2, and FD4 in the sepsis group were significantly elevated than that in the sham group, which indicated that the intestinal mucosal barrier function of sepsis rats was impaired. Moreover, upregulation of HIF-1α after treatment of DMOG cut down the levels of plasma D-lactic acid, DAO, FABP2, and FD-40; while downregualtion of HIF-1α after treatment of BAY 87–2243 aggravated the damage of sepsis to the structure and function of intestinal mucosa. suggesting that HIF-1α has a role in protecting the intestinal permeability and barrier function.

Oxidative stress is another key factor for intestinal mucosal barrier function disorder in sepsis. When the body is under severe stress, injury due to intestinal mucosal ischemia causes mucosal inflammation, which produces oxygen free radicals through xanthine oxidation dehydrogenase system [12], leading to necrosis and damage to the intestinal mucosal barrier function. MDA is the final product of lipid peroxidation and an important marker of oxidative stress [13]. In addition, the glutathione redox system within the critical antioxidant system in the body is obviously inhibited, reducing the ability of antioxidants to scavenge oxygen free radicals and eventually leading to the accumulation of a large number of toxic oxygen free radicals cells; this results in cellular lipid peroxidation, which leads to cell degeneration and eventually cell death [14]. CAT and SOD are antioxidant enzymes, and their activities reflect the ability of cells to scavenge reactive oxygen species and resistance to oxidative damage [15]. Under oxidative stress, CAT and SOD activities are significantly inhibited [13, 16]. In this study, CAT and SOD activities in the plasma of rats with sepsis were significantly decreased, while the MDA content was significantly increase, indicating that a large number of oxygen free radicals had damaged the mucosal tissue and that the antioxidant system in the body had been exhausted and inhibited, which could be partially reversed by the up-regulation of HIF-1α after the treatment of DMOG; while the down-regulation of HIF-1α after the treatment of BAY 87–2243. These findings suggest that HIF-1α plays a protective role against sepsis induced intestinal injury, possibly by inhibiting oxidative stress.

Inflammation also plays an important role in the pathophysiology of intestinal injury due to sepsis. Increases in the levels of TNF-α, IL-6 and IL-1β in the plasma indicated increased inflammation in the rats with sepsis. Among these factors, TNF-α plays a key role in the pathogenesis of sepsis, and IL-1β and IL-6 participate in the initiation of the sepsis cascade and reflect the degree of inflammation. Furthermore, TNF-α and IFN-γ can cause intestinal mucosal dysfunction in inflammatory bowel disease [17]. In current study, the increases of TNF-α, IL-6 and IL-1β were counteracted by up-regulation of HIF-1α induced by DMOG; while those inflammatory cytokines peaked in rats with down-regulation of HIF-1α induced by BAY 87–2243. These findings indicate that HIF-1α can inhibit intestinal inflammation, reducing the damage of inflammatory reaction to intestinal mucosa.

TJ proteins play an important role in maintaining intercellular connections and the cell barrier. Among TJ proteins, occludin is involved in adhesion and maintains the physical properties of the intestinal tract. Claudin-1 forms an ion-selective channel in cells that affects permeability to intercellular substances [18] while the TJ protein ZO-1 binds a variety of cytoskeletal proteins and plays a role in supporting the cytoskeleton [19]. In this study, the expression of the TJ proteins ZO-1, occludin and claudin-1 in the intestinal mucosa was decreased in septic rats, and the pathological morphology of the intestinal mucosa was destroyed, suggesting that sepsis leads to the destruction of the morphology of intestinal mucosa. In septic rats, DMOG, up-regulated the expression of HIF-1α and TJ proteins in the intestinal mucosa and alleviated the intestinal mucosal injury. In contrast, BAY 87–2243 down-regulated the expression of HIF-1α and TJ proteins and aggravated the intestinal mucosal injury.

Under physiological condition, intestinal epithelial cells are exposed to an environment of "physiological hypoxia", which underlies the survival of intestinal symbiotic bacteria and promotes intestinal material transportation [20]. Inflammation increases oxygen consumption and vasoconstriction and decreases the oxygen supply, resulting in hypoxia of intestinal epithelial cell hypoxia [21]. In this study, the expression of HIF-1α in the intestinal mucosa was significantly increased in septic rats compared to rats in the sham group, suggesting that sepsis caused intestinal mucosal cell hypoxia, and a compensatory increase in HIF-1α expression in the mucosal cells to improve the tolerance to hypoxia and promote proliferation, and maintain the integrity of the mucosal barrier. In previous studies, an HIF-1α activator was shown to significantly reduce CLP-induced renal damage in mice [22]. In mice with colitis, AKB-4924, an HIF-1α activator, enhanced intestinal mucosal barrier function, but no protective effect on the intestinal mucosa was observed in HIF-1α- deficient mice, indicating that HIF-1α in the intestinal mucosa is a target of AKB-4924-mediated protection [23]. Intestinal HIF-1α knockout in mice aggravated alcohol-induced destruction of the intestinal mucosal barrier [24]. Knockout and overexpression experiments showed that HIF-1α plays an essential role in regulating claudin-1 expression at the gene level, revealing that claudin-1 may be an important target gene of HIF-1α. The abnormal expression of Claudin-1 in the HIF-1α-deficient intestinal mucosa led to an abnormal structure of TJ structure [25].

There are some limitations in this study. Firstly, both DMOG and BAY 87–2243 are the indirect regulators of HIF-1α expression. In fact, DMOG inhibits the proline-4-hydroxylase and thus prevents HIF-1α degradation, thereby up-regulating the level of HIF-1α, but also prevents the degradation of some key inflammatory proteins such as IKKβ. BAY 87–2243 inhibits mitochondrial production of ROS by blocking mitochondrial complex I, which thereby reduces hypoxia-induced HIF-1α activity. Therefore, their effects cannot be attributed solelyto regulation of the HIF-1α pathway. Further studies using HIF-1α gene knockout technology could clarify the protective effect of HIF-1α on intestinal mucosa more clearly. Sepsis induced intestinal mucosal barrier function injury in rats is mainly characterized by increased permeability, which can be evaluated by both direct and indirect tests [26]. In this study, indirect tests were used to assess the intestinal permeability via the presence of permeability markers in the plasma and histological assessment of mucosal morphology; while direct assay was only performed to measure FITC-dextran leakage from the gut to the blood (FD4) without measurement of intestinal resistance, which was also another shortcoming of the study.

The study suggests that up-regulation of HIF-1α may be a promising treatment for controlling sepsis. Indeed, there is controversy to whether activation/inhibition of the HIF-1α pathway has negative or positive effects on organs, depending on different organ being examined or even different stage of disease for the same organ. Hummitzsch et al. [27] demonstrated that remote ischemic preconditioning (RIPC) ameliorates the ischemia/reperfusion (I/R) to the intestinal injury in rats. The underlying mechanisms may involve HIF-1α protein expression and a decreased serum activity of a 130 kDa factor with gelatinase activity. Other evidences suggested that elevated HIF-1α levels in the early stages of sepsis/septic shock are detrimental to survival. HIF-1α loss in the myeloid system protects mice from LPS-induced death by blocking inflammatory response, hypotension and hypothermia [28]; while in a rodent model of abdominal sepsis, increased HIF-1α activity due to PHD3 deletion reduces survival through an overwhelming innate immune response [29]. Fitzpatrick et al. [30] showed that HIF-1α/-2α double knockout mice under medial cerebral artery I/R featured neroprotection 24 h after reperfusion. However, no protective effects were found in these mice when reperfusion period is extended to 72 h. Unfortunately, the present study only investigated the effect of HIF-1a activation/ inhibition on intestinal mucosal barrier for just for one time point, 24 hrs post-CLP. The regulatory role of HIF-1a in different organs on different stages of sepsis needs to be further clarified.

The results of this study may be limited to our preset treatment protocol and detection time points, where HIF-1α shows a role in protecting the integrity of intestinal mucosa. The previous study by Fitzpatrick and his team [30] demonstrated that HIF-1α played an important time-dependent role during sepsis; regulating the inflammatory response during the acute stage and the regulating protective response during the later stages. They also showed that deletion of HIF-1α had an acute protective effect on LPS-induced hypoglycemia. Furthermore, reduced glucose uptake was observed in the heart and brown fat, in a time dependent manner, following loss of HIF-1α [30]. Shah found that in colitis activation of HIF-1α leads to a protective response, while chronic activation of HIF-2α increases proinflammatory response, intestinal injury and cancer [31]. In this study, HIF-1α has a protective effect on sepsis induced intestinal mucosa 24 hours after CLP. However, further studies are needed to clarify the time-dependent and different effects of HIF-1α signal on intestinal mucosa during sepsis. The effect of HIF-2α on sepsis induced intestinal mucosal injury also remains to be clarified. HIF-1α inhibitors have been proposed to treat a variety of pathophysiological conditions [32]. In the process of LPS induced sepsis, if we can clarify the different effects of the time dynamics of HIF-1α response on intestinal mucosa, considering the interest in HIF-1α targeted therapy, a better understanding of the impact of its dynamic response can help to achieve more targeted drug therapy.

In conclusion, the structure and function of the intestinal mucosal barrier in septic rats were seriously damaged through a mechanism involving the inflammatory response and oxygen free radicals. Mucosal cells up-regulated HIF-1α expression through self-regulation. HIF-1a activation protected the intestinal mucosal barrier by reducing intestinal mucosal permeability and regulating Claudin-1, a TJ protein, and then alleviated the damage to the intestinal mucosal barrier caused by sepsis; while a HIF-1α inhibition had the opposite effects. These results suggest that HIF-1α has a protective effect against sepsis-induced intestinal mucosal injury at 24 h post CLP, However, whether there is time dependence and the specific mechanism need to be further clarified.

## Supporting information

S1 File (DOCX)

S1 Raw images (PDF)
