# Supplementary material for: The protective effects of HIF-1α activation on sepsis induced intestinal mucosal barrier injury in rats model of sepsis
Source: PLoS One. 2022 May 16;17(5):e0268445. doi: 10.1371/journal.pone.0268445 (PMC9109928; doi:10.1371/journal.pone.0268445)

Supporting information contenets:

Inflammation cytokines---2
Oxidative stress parameter------------------------------------3
Intestinal permeability markers-------------------------------4
The HIH-1a and TJS expression-----------------------------5
The images of the intestinal morphology-------------------6
WB bands of GAPDH---7
WB bands of HIF-1a---8
WB bands of ZO-1--9
WB bands of Occudin--10
WB bands of Claudin---11

TNF-a	pg/mL	pg/mL	pg/mL	pg/mL
	Sham	Sepsis	DMOG	Bay 87-2243
1	43.7598	369.0948	178.986	386.9714
2	106.5636	309.6858	176.7228	483.2848
3	80.5368	339.1074	147.867	387.9858
4	72.6156	388.332	235.0002	425.209
5	19.4304	292.7118	211.8024	428.8938
6	11.5092	304.5936	89.5896	

IL-6	pg/mL	pg/mL	pg/mL	pg/mL
	Sham	Sepsis	DMOG	Bay 87-2243
1	44.31084	141.11128	126.05858	212.43792
2	74.41624	242.08016	117.49012	238.60646
3	57.27932	188.81676	98.0374	276.58558
4	39.21608	171.67984	132.54282	244.65906
5	36.43712	212.20634	113.78484	293.20674
6	21.15284	170.9851	130.69018	

IL-1β	pg/mL	pg/mL	pg/mL	pg/mL
	Sham	Sepsis	DMOG	Bay 87-2243
1	15.05758	69.27712	46.1374	95.13916
2	14.60386	94.23172	24.5857	115.78342
3	21.40966	88.33336	46.81798	115.5983
4	20.72908	89.46766	60.8833	102.90322
5	17.7799	90.60196	48.17914	147.26614
6	12.33526	60.42958	42.28078	

CAT	uM	uM	uM	uM
	**Sham**	**Sepsis**	**DMOG**	**BAY 87-2243**
1	69.18725	17.96575	37.84275	4.44025
2	53.515	32.8735	47.01675	13.761
3	55.044	29.051	53.515	20.2285
4	47.399	18.73025	27.522	14.90775
5	56.573	29.8155	35.9315	3.67575
6	55.8085	35.167	37.07825	

MDA	U/mL	U/mL	U/mL	U/mL
1	**Sham**	**Sepsis**	**DMOG**	**BAY 87-2243**
2	2.36503	5.94634	3.99922	9.04894
3	2.19118	5.49433	3.16474	6.98082
4	2.49252	4.81052	2.35344	5.68065
5	2.49252	4.93801	3.33859	6.49036
6	2.23754	3.88332	3.83696	6.56387
	1.88984	5.63341	4.42805	

SOD	U/mL	U/mL	U/mL	U/mL
	**Sham**	**Sepsis**	**DMOG**	**Bay 87-2243**
1	90.2637424	63.338241	62.6780262	38.9102922
2	96.6565049	55.5126822	63.2362369	38.1766677
3	78.7295543	53.513374	75.5527519	24.9495233
4	74.5240858	51.1977489	73.4508135	23.8401359
5	71.4668861	45.6229376	72.7442092	32.0266618
6	74.0450775	55.7388828	68.8140436	

DAO	ng/mL	ng/mL	ng/mL	ng/mL
	**sham**	**sepsis**	**DEX**	**Bay87-2243**
1	5.79	185.88	48.37	161.56
2	15.4	142.33	58.57	123.12
3	18.22	165.13	66.05	129.06
4	28.91	190.37	84.61	169.89
5	14.71	192.04	108.91	156.68
6	7.59	126.48	100.74	

D-lactic	ng/mL	ng/mL	ng/mL	ng/mL
	**Sham**	**Sepsis**	**DMOG**	**BAY 87-2243**
1	41.9226134	48.8095424	44.224723	88.1440496
2	40.0731658	83.1019222	52.3597058	104.80294
3	24.1588632	88.5726658	63.8249876	85.6950288
4	36.3548708	81.213675	74.0874818	98.0397682
5	34.9968848	75.2126702	47.490356	90.0747376
6	37.6805238	83.2765204	51.6160468	

FABP2	ng/mL	ng/mL	ng/mL	ng/mL
	**Sham**	**Sepsis**	**DMOG**	**BAY 87-2243**
1	1.1899076	5.8100091	3.99075845	8.67976885
2	2.54989	5.95670075	4.31430265	9.5006065
3	2.6521338	7.55522845	3.84933455	7.9833792
4	2.65660805	6.40637235	4.4198658	11.8415543
5	2.0865089	5.5906571	4.4226077	11.8087882
6	1.55205445	5.95316535	2.8624666	

FD-4				
	Sham	Sepsis	DMOG	BAY 87-2243
1	12	30	20	32
2	13	26	22	38
3	7	24	16	32
4	6	27	20	29
5	9	22	16	30
6	8	21	16	

HIF-1α

	Sham	Sepsis	Sepsi +DMOG	epsis+BAY87-224
	1	1.706598553	2.998809668	0.541552707
	1	1.582368265	2.193431332	0.370099115
	1	1.772611195	3.106369279	0.257796764

ZO-1

	1	0.56127624	0.948457587	0.399666203
	1	0.633064773	0.790593945	0.415709586
	1	0.708412973	0.895495097	0.116622442

Occudin

	Sham	Sepsis	Sepsi +DMOG	epsis+BAY87-224
	1	0.582520036	0.724782619	0.459463804
	1	0.574109924	0.673385675	0.390193281
	1	0.65762827	0.868248905	0.360874064

Occudin

	Sham	Sepsis	Sepsi +DMOG	epsis+BAY87-224
	1	0.577419357	1.002160068	0.172717716
	1	0.631436918	0.839658559	0.443790324
	1	0.670009868	0.769134006	0.363809586

HE

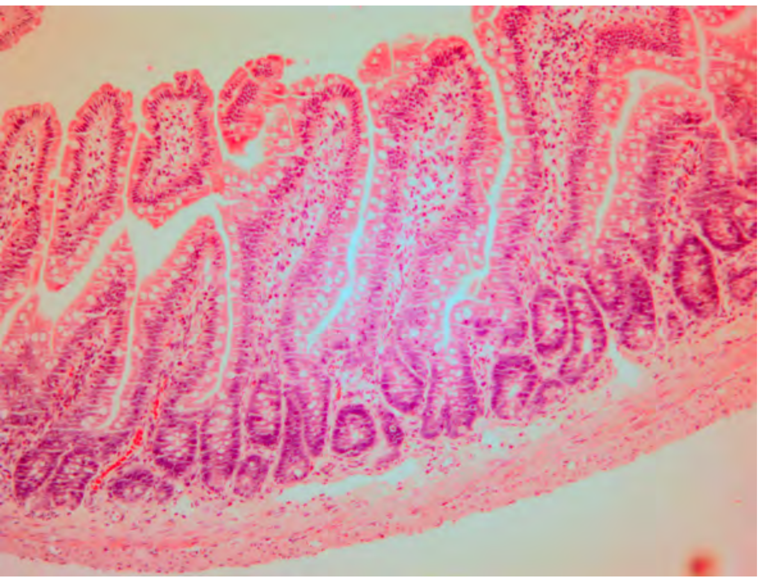

Sham 1

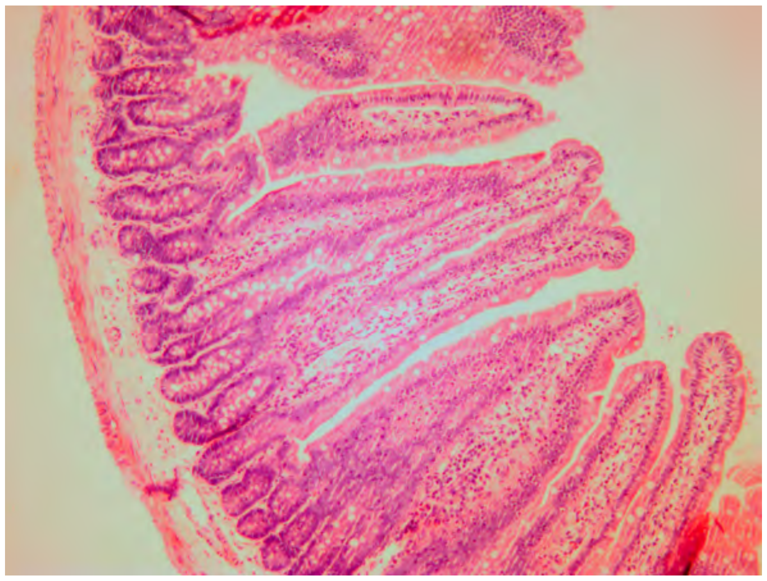

Sham 1

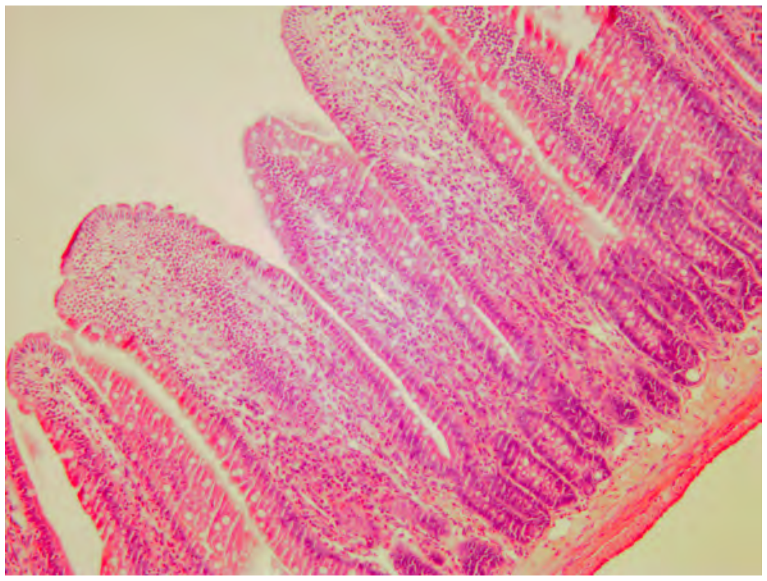

Sham 3

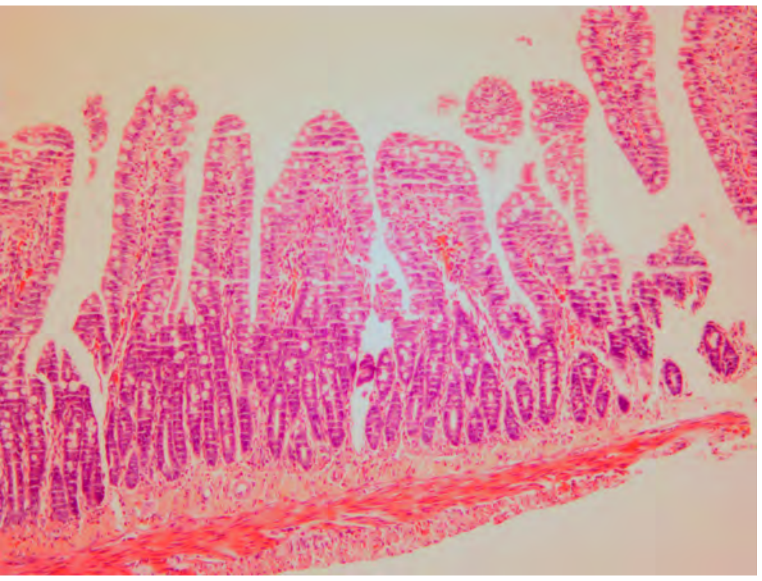

Sepsis 1

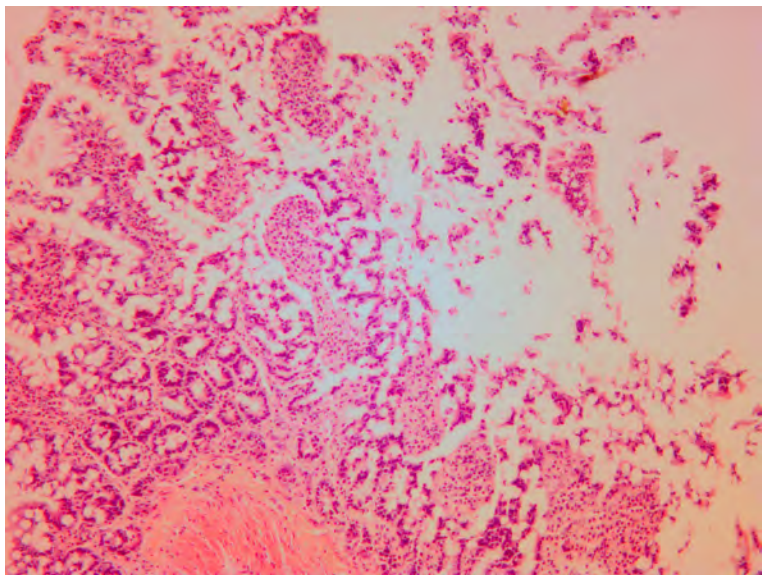

Sepsis 2

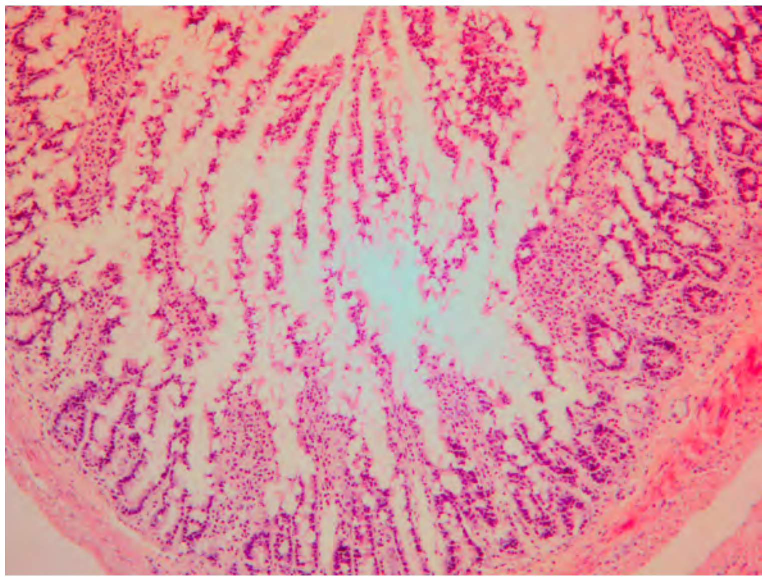

Sepsis 3

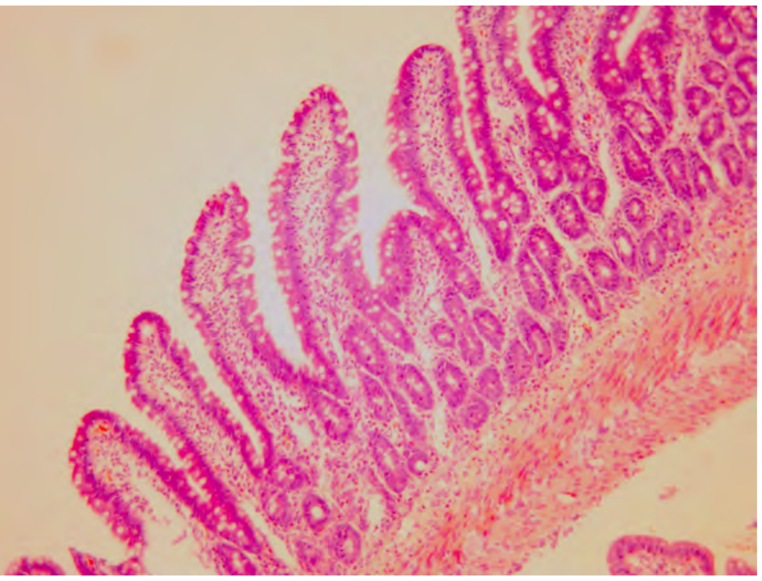

Sepsis +DMOG1

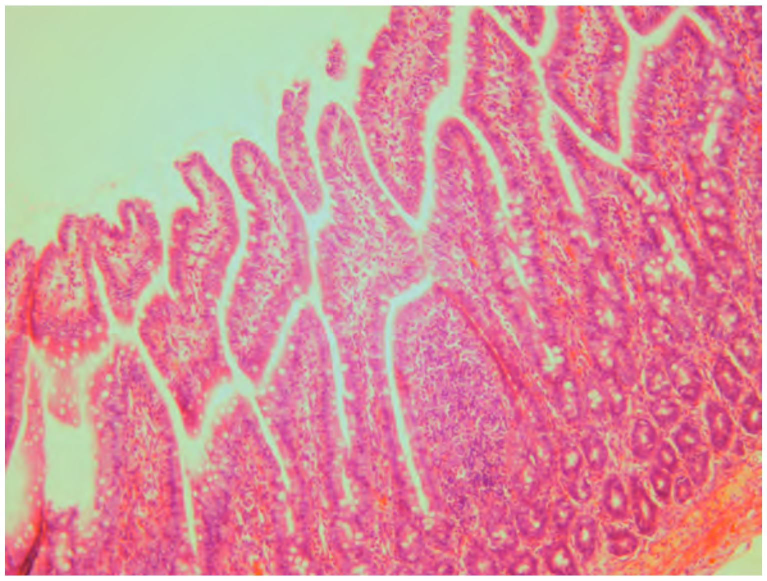

Sepsis + DMOG2

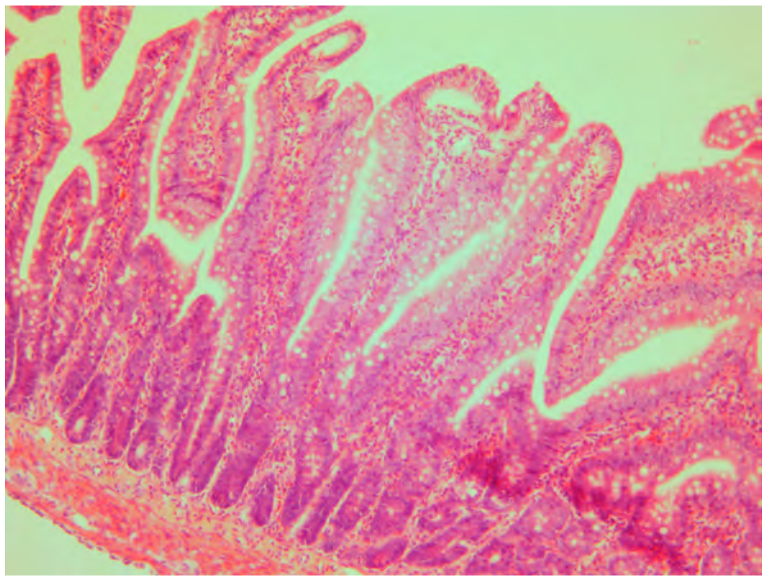

Sepsis +DMOG3

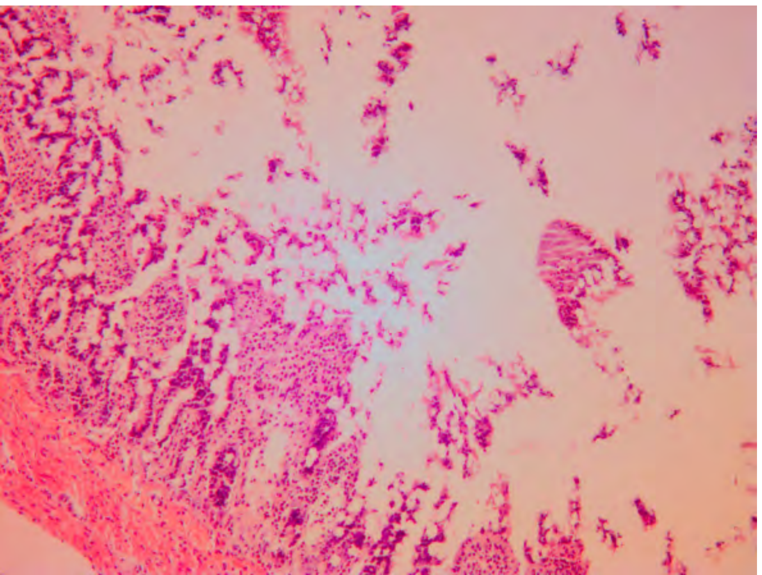

Sepsis + MAY 87-2243

1: GAPDH

Special note: it should be explained that the internal reference protein and the target protein come from the same film, but they are exposed separately.

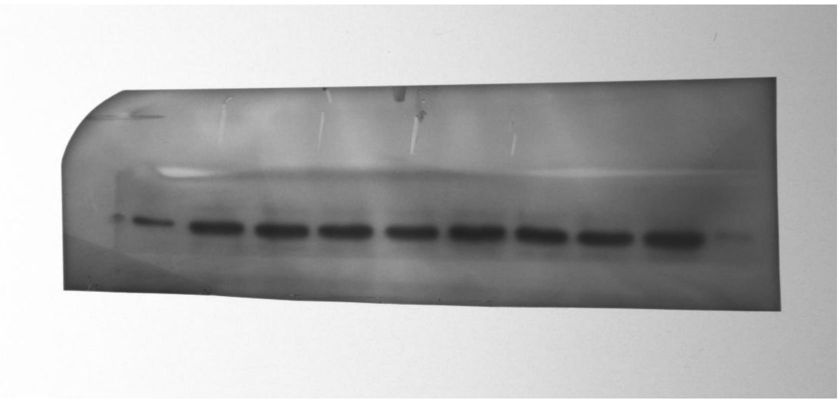

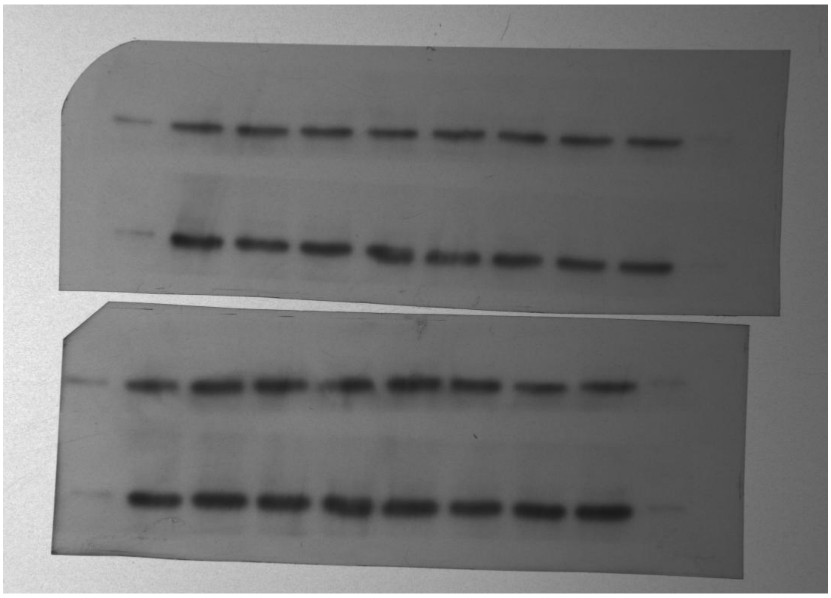

2: HIF-1α

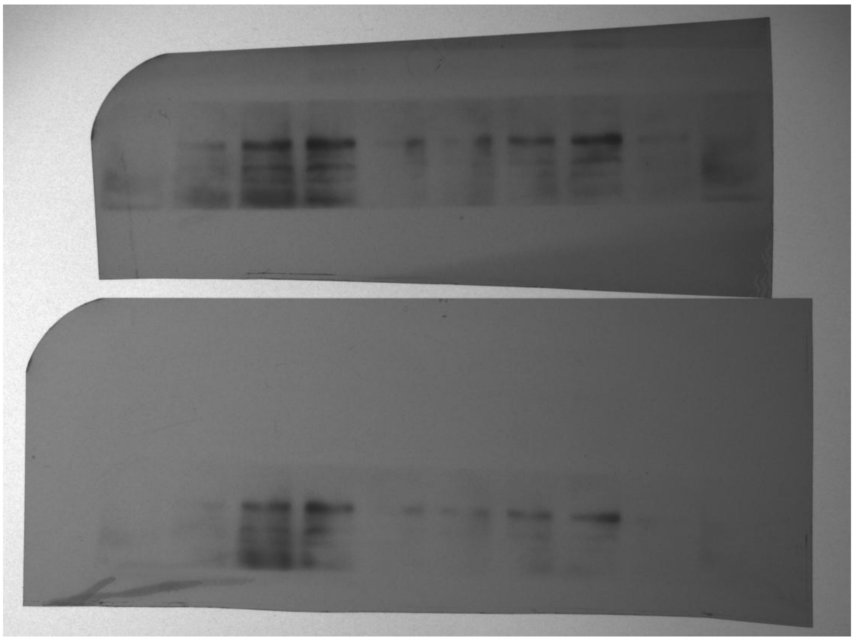

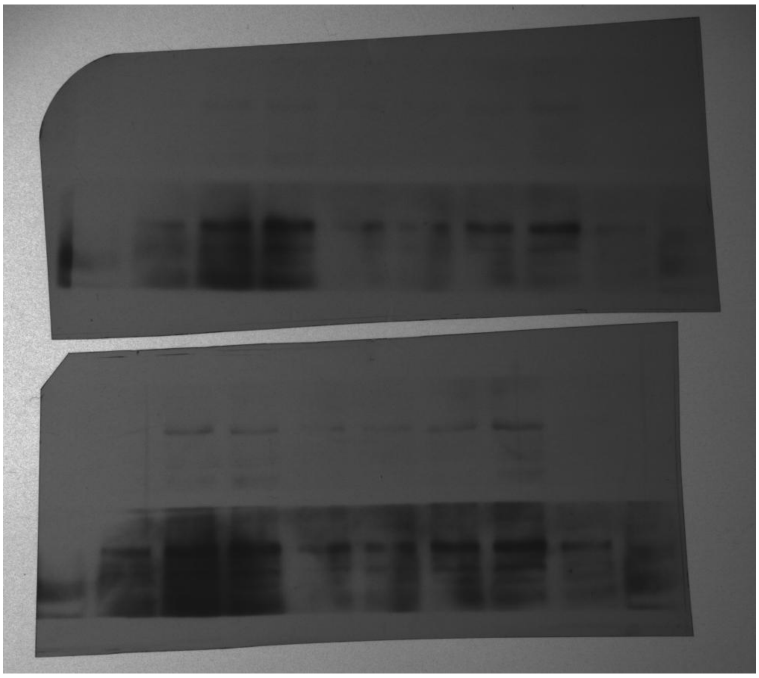

3: ZO-1

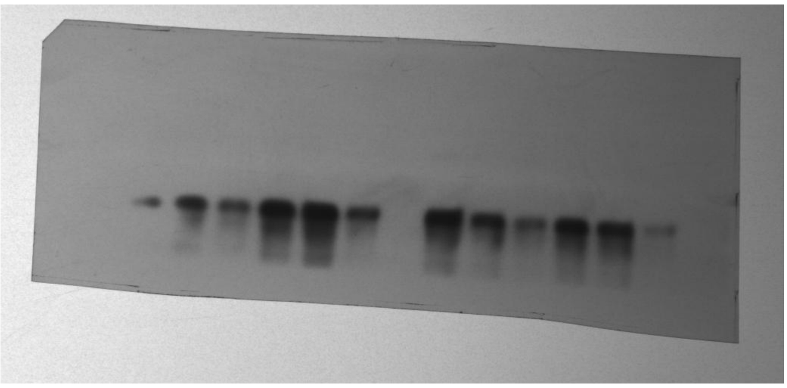

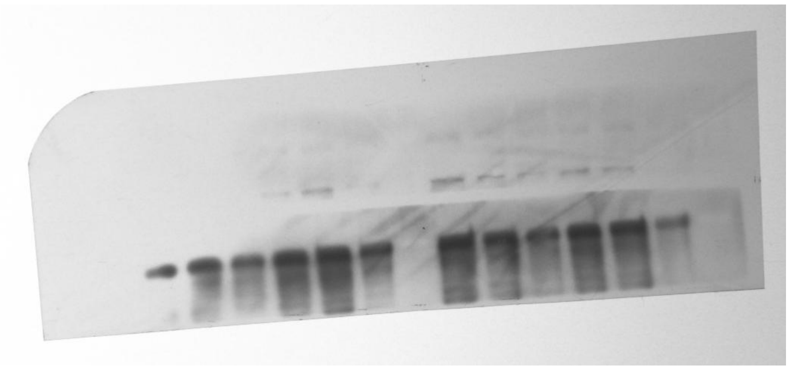

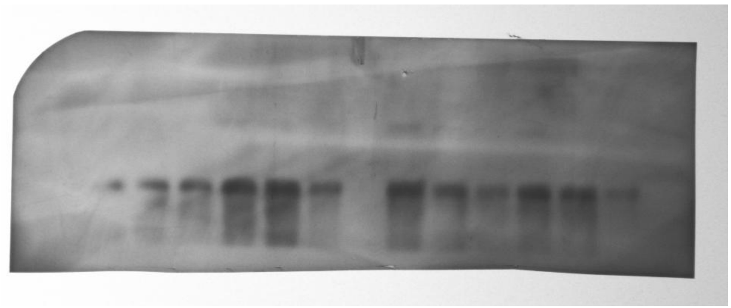

4: Occudin

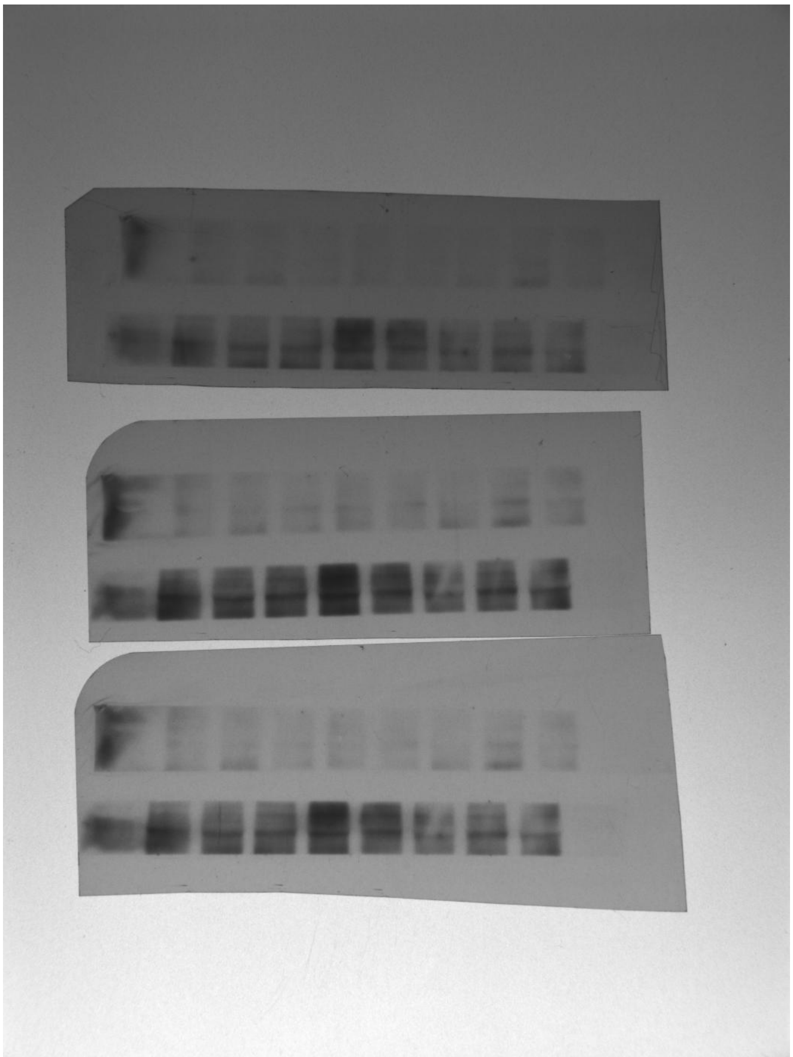

5: Claudin

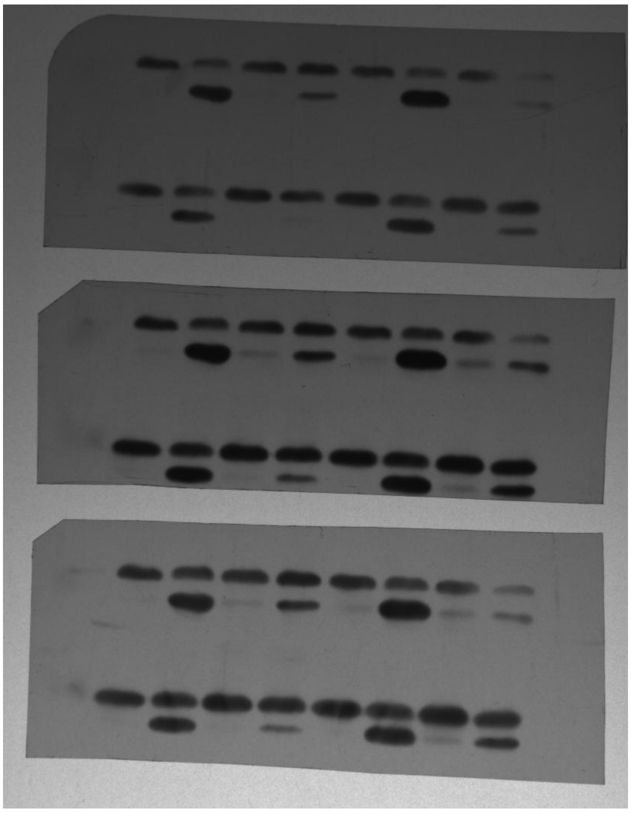

Supplement: S1 Raw images — (PDF) [file pone.0268445.s002.pdf]
