# Supplementary material for: The protective effects of HIF-1α activation on sepsis induced intestinal mucosal barrier injury in rats model of sepsis
Source: PLoS One. 2022 May 16;17(5):e0268445. doi: 10.1371/journal.pone.0268445 (PMC9109928; doi:10.1371/journal.pone.0268445)
Supplement: S1 File — (DOCX) [file pone.0268445.s001.docx]

PONE-D-21-16998R2

The protective effects of HIF-1α activation on sepsis induced intestinal mucosal barrier injury in rats model of sepsis

**Dear editor,**

**We have done some changes for the manuscript according to the JOURNAL REQUIREMENTS. All of changes are tracked in the submission files and our replies were highlighted in dark blue.**

**Regards,**

**Yuhong.**

Dear Dr. Li:

Thank you for submitting your work to PLOS ONE. Your manuscript will be formally accepted and enter production after you complete the requests below. Please note that you will not be able to make changes to your manuscript once it enters the production process. PLOS ONE does NOT provide author proofs. Any changes other than those requested in this email will need to be reviewed by the Academic Editor and reviewers; this will delay the formal acceptance of your manuscript.

To access your manuscript and complete these changes, please follow this link: [https://www.editorialmanager.com/pone/l.asp?i=49180079&l=APDU8WE6.](https://www.editorialmanager.com/pone/l.asp?i=49180079&l=APDU8WE6.)

Please note that this link should not be shared with other people to ensure that any action taken on this submission is done by you, the Corresponding Author.

You will find the submission in "Current Task Assignments." From there you must download your submission files via the "Assignment Files" link. You can also download the manuscript file attached to this email; the document has been formatted to track all changes, and we will only accept this version with locked tracked changes returned to us.

To opt in or out of publishing your peer review history, please answer the Peer Review History question within this task. Please note we are unable to send your article to production without this.

**Author reply: Our choice is to opt out of publishing the peer review history.**

Once you have made all the required changes, click "Submit Task" to upload the corrected files.

Your task is due May 07 2022 11:59PM.

Please contact plosone@plos.org with any questions or concerns. For billing related questions, please contact our Author Billing department directly at authorbilling@plos.org. For questions regarding your press release or the press process, please contact onepress@plos.org

With kind regards,

PLOS ONE staff

JOURNAL REQUIREMENTS:

1. Please ensure that the author list and affiliations are correct on the title page of your manuscript, and that your author contributions, competing interests, and financial disclosure are correct as listed below. All of these sections will be indexed in PubMed and published by PLOS ONE as you have written them. Please email plosone@plos.org if any changes to this content need to be made.

Xiuzheng Lei: (the right is Xiuzhen Lei)

Data curation

Wenbin Teng:

Investigation

Resources

Writing – original draft

Ying Fan:

Investigation

Resources

Yeke Zhu:

Investigation

Resources

Shengmai Zhu:

Conceptualization

Methodology

Liuxu Yao:

Investigation

Yuhong Li:

Conceptualization

Data curation

Formal analysis

Funding acquisition

Methodology

Project administration

Supervision

Validation

Visualization

Writing – review & editing

**Author reply: Except the first author, the other authors are right. We have sent an email plosone@plos.org for this change.**

Please see here for the full list and definition of contributor roles: [http://journals.plos.org/plosone/s/authorship#loc-author-contributions](http://journals.plos.org/plosone/s/authorship#loc-author-contributions)

Please ensure that the Competing Interests and Financial Disclosure statements listed below are suitable for publication. These sections will be indexed in PubMed and published by PLOS ONE as you have written them. Please email plosone@plos.org if any changes to these statements need to be made.

Competing Interests: The authors have declared that no competing interests exist.

**Author reply: It is right.**

Financial Disclosure: This study was supported by Zhejiang Provincial Department of Science and Technology Fund (grant number LY21H150001 and LGF19H030011), Zhejiang Provincial Health Committee Fund (grant number 2020KY329). The funders had no role in study design, data collection and analysis, decision to publish, or preparation of the manuscript.

**Author reply: It is right.**

2. To prevent production delays, we recommend using the Author Formatting Checklist to confirm that your paper meets PLOS ONE's typesetting requirements for References, Tables, and Figures: [http://journals.plos.org/plosone/s/file?id=c819/plos-one-author-formatting-checklist.docx.](http://journals.plos.org/plosone/s/file?id=c819/plos-one-author-formatting-checklist.docx.)

This checklist is a reference tool for you; please do not upload the completed Author Formatting Checklist with your submission files.

3. To ensure your figures meet our technical requirements, please run each figure included in your submission files through the PACE tool: [https://pacev2.apexcovantage.com/.](https://pacev2.apexcovantage.com/) PACE will assess whether your figures meet our technical requirements and will fix the figure(s) or identify any problem(s) that cannot be automatically fixed. It can also convert figures to TIFF format, resize, and rename figures to meet our naming conventions.

To use PACE, first register as a user. Follow the instructions on the site for assessing and converting your figure files. If you experience any difficulty using this tool or have questions about any of the figures and/or images in your paper, please inform the journal office in your response letter.

**Author reply: I have run each figure included in our submission files through the PACE tool smoothly.**

CONFIDENTIAL: This email and any attachments are confidential and for the sole use of the individual(s) to whom they are addressed. If you have received this message in error please delete the message and notify plosone@plos.org.
